# Bearing fault diagnosis based on particle swarm optimization fusion convolutional neural network

**DOI:** 10.3389/fnbot.2022.1044965

**Published:** 2022-11-25

**Authors:** Xian Liu, Ruiqi Wu, Rugang Wang, Feng Zhou, Zhaofeng Chen, Naihong Guo

**Affiliations:** ^1^School of Information Technology, Yancheng Institute of Technology, Yancheng, China; ^2^Yancheng Xiongying Precision Machinery Company Limited, Yancheng, China

**Keywords:** CNN, PSO, fault diagnosis, adaptive adjustment, bearing

## Abstract

Bearings are the most basic and important mechanical parts. The stable and safe operation of the equipment requires bearing fault diagnosis in advance. So, bearing fault diagnosis is an important technology. However, the feature extraction quality of the traditional convolutional neural network bearing fault diagnosis is not high and the recognition accuracy will decline under different working conditions. In response to these questions, a bearing fault model based on particle swarm optimization (PSO) fusion convolution neural network is proposed in this paper. The model first adaptively adjusts the hyperparameters of the model through PSO, then introduces residual connections to prevent the gradient from disappearing, uses global average pooling to replace the fully connected layer to reduce the training parameters of the model, and finally adds a dropout layer to prevent network overfitting. The experimental results show that the model is under four conditions, two of which can achieve 100% recognition, and the other two can also achieve more than 98% accuracy. And compared with the traditional diagnosis method, the model has higher accuracy under variable working conditions. This research has important research significance and economic value in the field of the intelligent machinery industry.

## Introduction

In the operation of machinery, bearings are often one of the most easily damaged parts due to the high frequency of use. And because the bearing generally plays the role of supporting the main shaft and transmitting the torque, it plays a decisive role in whether the equipment can work normally. Once the equipment fails, it may lead to catastrophic consequences, so it is particularly important for bearing fault diagnosis (Liu, 2017; Du et al., 2020; Li et al., 2020; Yu et al., 2020). When the bearing fails, the vibration signal generated has certain characteristics. The fault diagnosis of the bearing is to classify the collected vibration signal. The traditional diagnosis process is to extract relevant features from the collected vibration signals, and then use a specific classifier to classify and identify. For example, In 2020, researchers such as Yang et al. (2020) used wavelet packets to extract bearing fault features and input the obtained feature information into an improved Bayesian classification model for classification. The experimental results show that compared with the model before optimization, the modeling time is shorter and the fault diagnosis accuracy is higher. In 2020, researchers such as Wang et al. (2020) proposed a rolling bearing fault diagnosis method based on minimum entropy deconvolution (MED) and Autogram. This method removes noise through MED, and can effectively highlight fault features while obtaining the best frequency band. Compared with the existing methods at that time, it can detect the demodulation frequency band and fault frequency more accurately, highlight the fault characteristics and improve the fault detection effect. In 2021, researchers such as Zhen et al. (2021) proposed a bearing fault signal feature extraction method based on the combination of wavelet packet energy and kurtosis spectrum. This method can clearly obtain the fault characteristic frequency and its higher harmonics.

It can be seen from the existing research results that this kind of bearing fault diagnosis method can effectively extract features and classify them. However, it is necessary to manually extract features, which requires a large workload and subjective factors. Therefore, it is limited in practical application. In order to solve these problems, the study of deep learning in bearing fault diagnosis has attracted extensive attention of researchers. Deep learning is an end-to-end recognition method, which can extract features adaptively, and better solve the defects of manual feature extraction. In 2018, researchers such as Qu et al. (2018) proposed a fault diagnosis algorithm based on an adaptive one-dimensional convolutional neural network. Features are extracted through the convolution and pooling layers of the convolutional neural network, and classified through the Softmax layer. In 2020, researchers such as Gu et al. (2020) proposed an adaptive one-dimensional convolutional neural network and long short-term memory network fusion bearing fault diagnosis method. This method improves the accuracy as well as the validity and stability of the model. In 2021, researchers such as Liu et al. (2021) proposed a fault diagnosis method for rolling bearings based on parallel 1DCNN. Improve the ability of fault diagnosis by fusing time domain and frequency domain features, This method can make full use of the extracted time domain and frequency domain feature information, and has better fault diagnosis ability.

Judging from the existing research progress, the research on bearing fault diagnosis based on deep learning has achieved good results, which can achieve accurate identification of bearing fault diagnosis. However, the generalization and robustness of the existing diagnostic models still need to be improved. In view of these research problems, this paper designs a convolutional neural network model based on particle swarm optimization fusion. Optimizing the network’s hyperparameter learning rate through PSO (Particle Swarm Optimization) enables the network to achieve a better gradient global minimum in the gradient descent process, And introduce residual connections to alleviate gradient disappearance, Then use global average pooling to replace part of the fully connected layer to reduce the amount of parameters and improve generalization, Finally, a Dropout layer is added to prevent the network from overfitting.

## Analysis of particle swarm optimization fusion convolutional neural network algorithm

Convolutional neural networks have the characteristics of end-to-end, local perception and parameter sharing. Feature extraction is performed on the input data through multiple filters. When the network is deepened, the extracted features are also more advanced, and robust features with shift invariance are obtained in the original data. Compared with models that extract features manually, convolutional neural networks have stronger discriminative and generalization capabilities. It can effectively obtain the local features of the data to be tested, and is widely used in classification problems such as image processing, speech recognition, and natural language processing (Maite et al., 2020; Tian, 2020; Wang et al., 2021; Chen et al., 2022; Sultana et al., 2022).

Convolutional neural networks are composed of convolutional layers, pooling layers, fully connected layers and output layers. The convolutional layer and the pooling layer perform feature extraction on the input data. The mathematical model of the convolution operation of the convolution layer can be expressed as:


(1) $$y_{\mathrm{ij}}=f\,\left(\sum_{i=1}\sum_{j=1}x_{\mathrm{ij}}\,w_{\mathrm{ij}}+b\right)$$

Among them, *y*_*ij*_ is the output, *x*_*ij*_ is the input, *w*_*ij*_ is the weight value, *b* is the bias, and *f*() is the activation function. The purpose of the activation function is to make the input not a linear function so that it can approximate any function and make the network generalization ability stronger. The activation function generally adopts the Relu function and its mathematical expression is:


(2) f⁢(x)=max⁢(0,Y)

Among them, *f*(*x*) is the output, and *Y* is the activation value that the convolutional addition will add the bias value.

The pooling layer is mainly divided into maximum pooling and minimum pooling, and its mathematical models can be described as:


(3) $$y_{\max}=\max\,\left(x_{11},\ldots \,x_{\mathrm{ij}}\right)$$

(4) $$y_{\mathrm{mean}}=\mathrm{mean}\,\left(x_{11},\ldots \,x_{\mathrm{ij}}\right)$$

**FIGURE 1:**
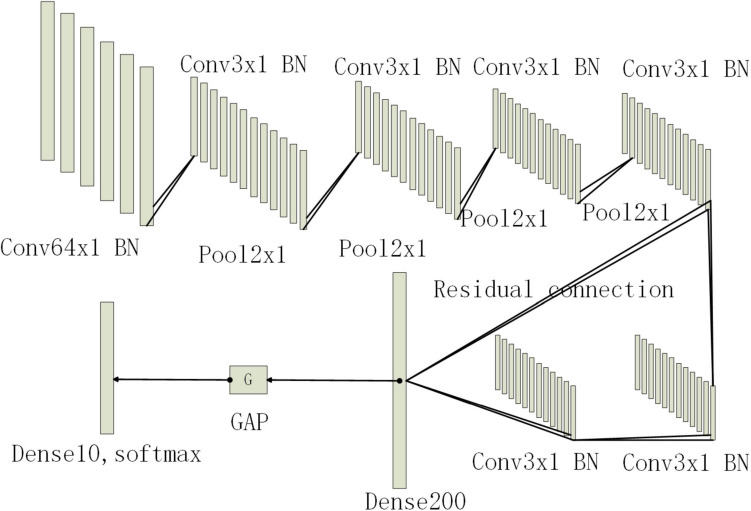
Network structure diagram.

Among them, *y*_*max*_ and *y*_*mean*_ is the pooling output.*x*_*ij*_ is the output value at position (i, j) that take the maximum value or average value in the pooling area for output. After multiple convolution and pooling layers, the input fully connected layer fuses the learned features and maps them to the label space. Finally, the classification results are output through the Softmax function in the output layer. Since the bearing fault data is one-dimensional amplitude data, this paper will use the backbone of the convolutional neural network as a one-dimensional convolutional neural network. The convolution kernel is a one-dimensional structure, and the output of each convolutional layer and pooling layer will be a one-dimensional feature vector. The first layer of the convolutional layer uses large convolution kernels and large steps to increase the field of view of this layer, which can effectively extract the overall timing features. Then, a small convolution kernel is used to deepen the network. In order to avoid the risk of gradient disappearance as the network deepens, this paper will introduce a residual structure. Then replace some of the fully connected layers with global average pooling. Compared with the fully connected layer, the global draw pooling can greatly reduce the training parameters and speed up the training speed, while enhancing the generalization of the network and preventing overfitting. A BN operation is added to each convolutional layer. It can play the function of controlling the gradient explosion or gradient disappearance. Finally, a Dropout layer is added to prevent the network from overfitting. Its network structure is shown in Figure 1, Table 1. Conv represents the convolution layer, Pool represents the pooled layer, Dense represents the fully connected layer, BN represents the batch normalization layer, and GAP represents the global average pooled layer. The following numbers represent the convolution core size and x1 represents the one-dimension.

**TABLE 1:** Model structure parameters.

Layers	Layers size	Quantity	Output size
Input	–	–	2048 × 1
Conv_first	64	16	128 × 16
Pool1	2	16	64 × 16
Conv2	3	32	64 × 32
Pool2	2	32	32 × 32
Conv3	3	64	32 × 64
Pool3	3	64	16 × 64
Conv4	3	64	16 × 64
Pool4	2	64	8 × 64
Conv5	3	64	8 × 64
Conv6	3	64	8 × 64
Dense	1	200	8 × 200
Dropout	1	200	8 × 200
GAP	–	200	1 × 200
Dense10	–	10	1 × 10
softmax	–	–	1 × 10

In order to improve the performance, when training the convolutional neural network, it is necessary to set certain hyperparameters for the network. If the hyperparameters are manually set, it will take a long time and lack generalization to different scenarios. Due to its advantages of easy convergence and strong global search ability, particle swarm optimization method is very suitable for hyperparameter optimization of machine learning algorithms (Shao et al., 2020; Li et al., 2022). The training of the convolutional neural network model is the process of finding the lowest point of the gradient. In order to better obtain the lowest point of the global gradient, the setting of the learning rate is particularly important. When the learning rate is set too large, it will miss the global optimal solution or fail to converge at all, and a better training structure cannot be obtained. When the learning rate is set too small, the convergence of the model will be very slow, and the model may be unable to jump out of the local optimal solution. The artificial setting of the learning rate will be objective, and it is impossible to give a good value in different model use cases. Therefore, this paper introduces the PSO particle swarm algorithm to set an adaptive setting for the hyperparameter learning rate.

**FIGURE 2:**
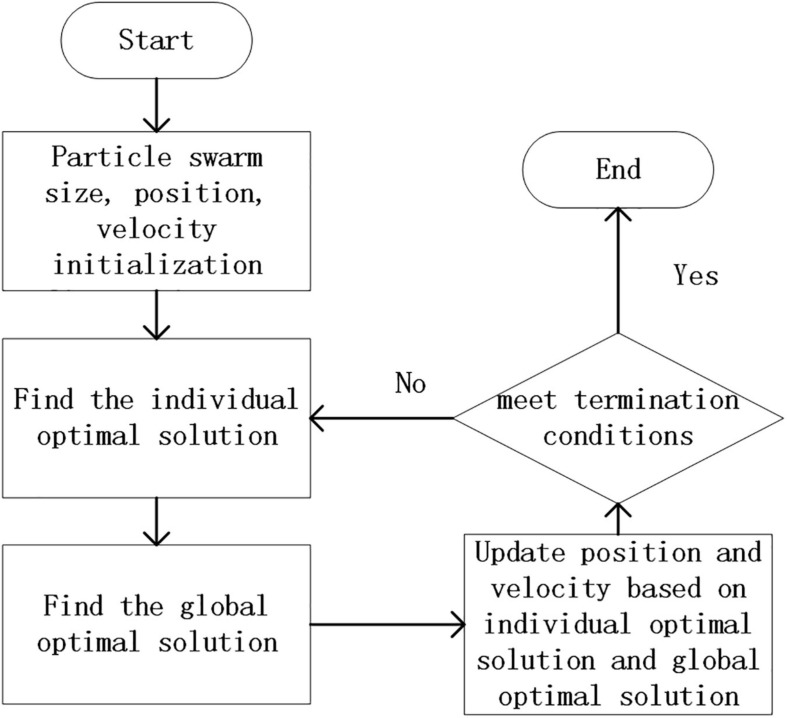
Particle swarm optimization flowchart.

Particle swarm optimization is to generate a group of particles in the space that needs to be solved, and each particle has two attributes of velocity and position. Among them, the speed represents the speed of the movement, and the position represents the direction of the movement. Each particle searches for the optimal solution individually in space, and records it as the current individual best solution to obtain the local optimal solution *P*_*best*_, and then shares all individual extreme values with other particles in the entire particle swarm. A global optimal solution *G*_*best*_ is selected from the individual optimal solutions of the particle swarm, and all particles adjust their speed and position according to the individual optimal solution *P*_*best*_ and the global optimal solution *G*_*best*_. Its process is shown in Figure 2.

The update formula of particle position and velocity of particle swarm is:


(5) $$\left\{ \begin{array}{c} V_{\mathrm{id}}=w\,V_{\mathrm{id}}+C_{1}\,r\,a\,n\,d\,o\,m\,\left(0,1\right)\,\left(P_{\mathrm{id}}-X_{\mathrm{id}}\right)\\ +C_{2}\,r\,a\,n\,d\,o\,m\,\left(0,1\right)\,\left(P_{\mathrm{gd}}-X_{\mathrm{id}}\right)\\ X_{\mathrm{id}}=X_{\mathrm{id}}+V_{\mathrm{id}} \end{array}\right.$$

**FIGURE 3:**
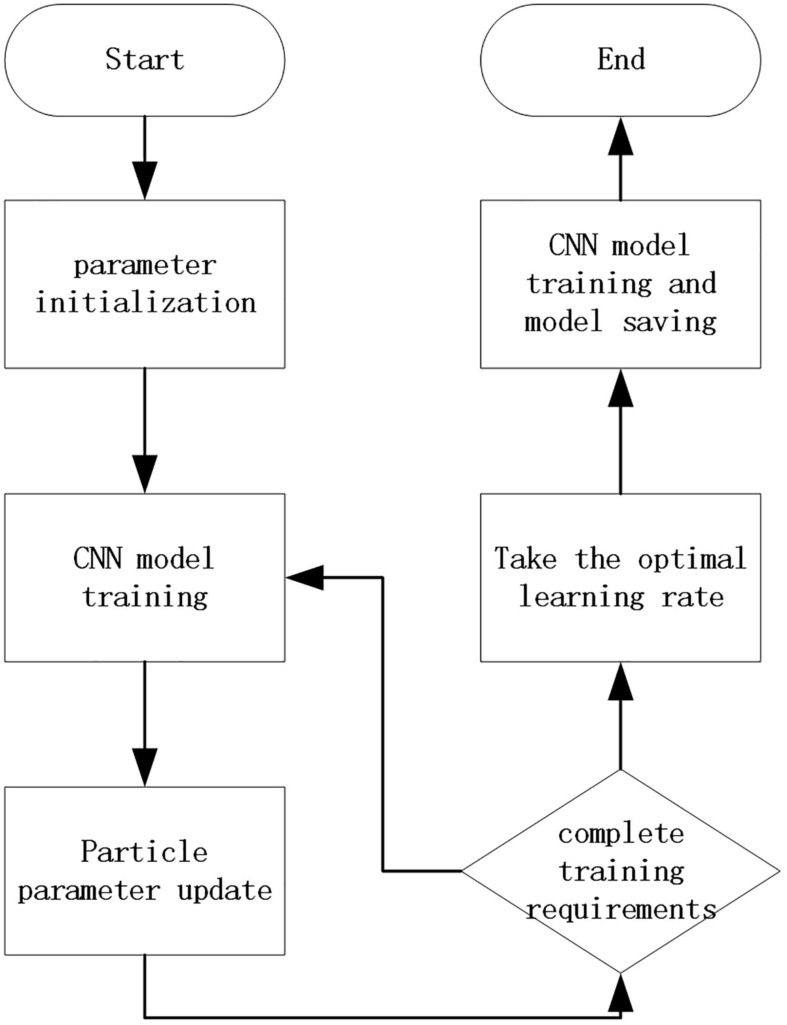
Overall model flowchart.

Among them, *w* is the inertia factor, *C*_*1*_ and *C*_*2*_ are acceleration constants, generally *C*_*1*_ and *C*_*2*_ are numbers between 0 and 4, and *random*(0,1) is a random number between 0 and 1. *P*_*id*_ represents the d-th dimension of the individual extreme value of the i-th variable, *P*_*gd*_ represents the d-th dimension of the global optimal solution, and *X*_*id*_ is the current position. The concrete implementation process is shown in Figure 3. Before adding particle swarm optimization, complete construction of the improved network is carried out first. Then, the parameters of particle swarm optimization were set. In this paper, when using particle swarm optimization to optimize the learning rate, the parameters set as the number of particles is five, the number of iterations of the optimization algorithm is 20, the range of the optimized parameters is (0.001, 0.00001), and the maximum moving speed each time is 0.0005. In the process of algorithm operation, the optimal solution of particle swarm optimization is constantly updated according to a verification accuracy rate of the current network, and finally an optimal value will be output at the end of the iteration times of the particle swarm optimization algorithm. After receiving the optimal value, the network will train the network with the optimal value of learning rate to obtain the network model of particle swarm optimization fusion convolutional neural network.

Compared with the traditional convolutional neural network algorithm, the particle swarm optimization fusion convolutional neural network algorithm can train the model more effectively, and the trained model has higher accuracy and generalization. It is mainly through particle swarm optimization to adaptively select the hyperparameter of the learning rate, because particle swarm optimization is a random evolution method to obtain the optimal value. Therefore, the selected hyperparameters can be more suitable for the current network model, so that the model can obtain a better solution during gradient descent. Compared with the ordinary convolutional neural network in terms of network structure, large-scale and large-step convolution kernels are used in the first layer to improve the overall characteristics of the network data. Secondly, while deepening the network, residual connections are used to prevent the gradient of the network from disappearing. Then, in the fully connected part of the ordinary network, the global average pooling is used to replace part of the fully connected layer, which improves the generalization of the model and reduces the amount of some parameters. Finally, a Dropout layer is used to prevent the network from overfitting.

## Experiment and result analysis

### Dataset

The dataset used in this paper is the public bearing dataset of Case Western Reserve University. The data with the sampling frequency of 12 kHz in the data set is selected, and it is the vibration acceleration signal data of the bearing SKF620 under different working conditions and different faults. Among them, the working conditions are that the different bearing speeds are 0 horsepower (HP) (1,797 r/min), 1HP (1,772 r/min), 2HP (1,750 r/min), and 3HP (1,730 r/min). The dataset can be divided into nine fault types and one normal type in total. The fault types are rolling element fault, outer ring fault, inner ring fault, etc. Each fault type contains three fault sizes of 0.007, 0.014, and 0.021 in, plus the sampling data of the normal state. Each sample is a continuous 2,048 data points, and there are 10,000 samples in each working condition. This paper divides 10,000 samples with a ratio of 0.7, 0.2, and 0.1, which are training set, validation set, and test set, respectively. The specific experimental samples are shown in Table 2.

**TABLE 2:** Dataset summary.

Fault type	Size/In	Number of train	Number of value	Number of test	Logogram
Normal	0	700	200	100	Normal
Rolling element	0.007	700	200	100	B007
	0.014	700	200	100	B014
	0.021	700	200	100	B021
Inner ring	0.007	700	200	100	OR007
	0.014	700	200	100	OR014
	0.021	700	200	100	OR021
Outer ring	0.007	700	200	100	IR007
	0.014	700	200	100	IR014
	0.021	700	200	100	IR021

### Experimental platform and model parameters

The experiment was implemented in the Tensorflow2.0-GPU environment, the programming language was Python, and the computer was configured with a windows10 system, Intel Core i5-9300H processor, 32G memory, and NVIDIA GTX1660Ti graphics card. The ratio of training set, validation set, and test set is 7:2:1. PSO particle swarm optimization is used to optimize the learning rate. Adam optimizer is used for training. The number of particles in the PSO particle swarm optimization operation is five. The number of algorithm iterations is 20, the maximum value of the optimized parameters is 0.001, the minimum value is 0.00001, and the maximum moving speed of the particles is 0.005 each time. After obtaining the optimized learning rate, the network is trained for 20 iterations, each batch size is eight, and the loss function adopts the cross-entropy loss function.

### Analysis of experimental results

The working conditions of the bearing fault data set are 0HP (1,797 r/min), 1HP (1,772 r/min), 2HP (1,750 r/min), and 3HP (1,730 r/min) for different bearing speeds, respectively. The datasets under four working conditions are divided into training set, validation set, and test set with a ratio of 7:2:1. The improved adaptive one-dimensional convolutional neural network model is trained through training set and validation set to obtain network models under different working conditions. Then use the test set to test the network model to obtain the recognition accuracy of the model under each working condition. Then the one-dimensional convolutional neural network model 1DCNN (Li et al., 2020) and the adaptive one-dimensional convolutional neural network PSO-1DCNN are trained and tested in the same way, and the traditional method SVM (Shun et al., 2021) is introduced for experimental comparison. PSO-1DCNN adds the PSO optimization proposed in this paper on the basis of 1DCNN. However, the network structure is not optimized. The results are shown in Table 3.

**TABLE 3:** Comparison of bearing fault diagnosis results between different models.

Models	Accuracy (×100%)			
	0HP	1HP	2HP	3HP
SVM	70.79	72.8	73.6	72.2
1DCNN	98.9	97.5	98.8	99.3
PSO-1DCNN	99.2	98.2	99.4	99.5
Model of this paper	99.9	98.8	100	100

**FIGURE 4:**
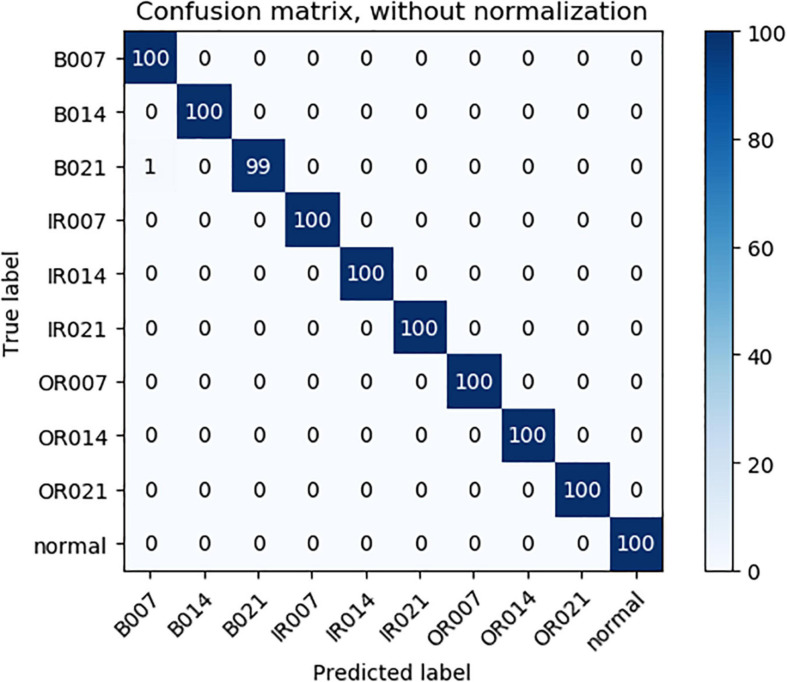
0HP Test results confusion matrix.

**FIGURE 5:**
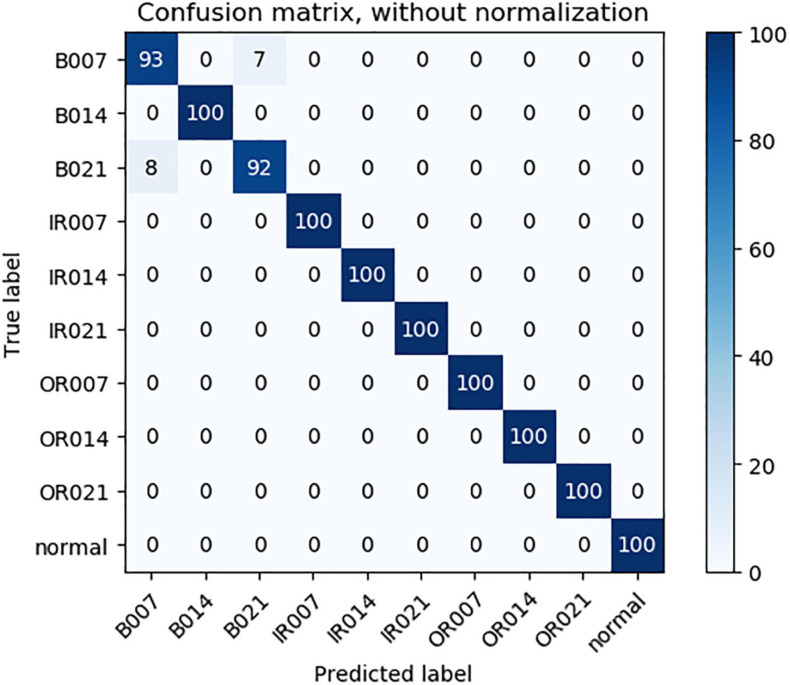
1HP Test results confusion matrix.

As can be seen from Table 3, compared with 1DCNN, PSO-1DCNN, and SVM, the model proposed in this paper can better identify faults. Moreover, the recognition degree reached 100% under the working conditions of 2HP and 3HP, and at the same time, the recognition degree under the 0HP working condition was also close to 100% and reached 99.9%. In order to better analyze the experimental results, a confusion matrix is introduced to analyze the experimental results under 0HP and 1HP in detail. The confusion matrix of 0HP and 1HP test results is shown in Figures 4, 5, in which the vertical axis is the real label, and the horizontal axis is the predicted result. The label name corresponds to the fault type as shown in Table 1. It can be seen from the figure that the model has misidentified the fault identification of B007 and B021, and the model under the 0HP condition incorrectly identified 1% of the B021 type faults in the test set as B007 faults. The model under the 1HP condition incorrectly identifies 7% of the B007-type faults and 8% of the B021-type faults in the test set as B021-type faults and B007-type faults, respectively, while the remaining nine state types can be 100% identified. It shows that the model in this paper has a high fault identification degree.

#### T-distributed stochastic neighbor embedding visualization

**FIGURE 6:**
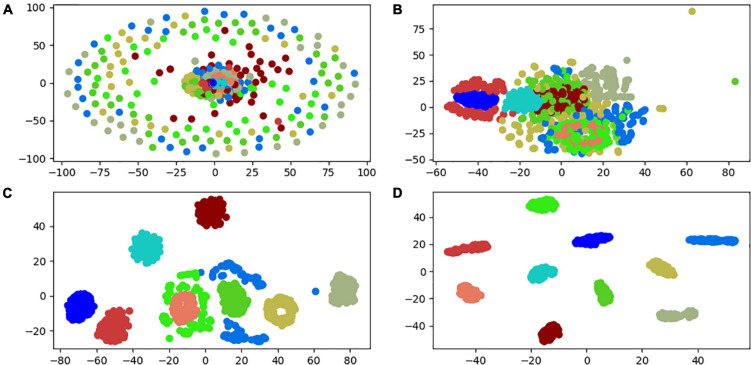
Partial layer classification result visualization. **(A)** Input layer. **(B)** First convolutional layer. **(C)** Intermediate convolutional layer. **(D)** Output layer.

In order to further check the classification ability of each layer, the t-SNE (t-distributed stochastic neighbor embedding) dimension reduction algorithm in manifold learning is introduced (Caio et al., 2021). To visualize the first and middle layers of the input vector of the 3HP model and the final fully connected layer in 2D. The experimental results are shown in Figure 6. Among them, a small dot of each color indicates a type of failure. As can be seen from the figure, the initial input vector is a disorganized vector distributed in the vector space. After passing through the first convolutional layer, the small dots of the same color begin to gather. After passing through the intermediate layer classification, most of the samples have been clustered in the form of the same fault. However, there are still two types of faulty samples that are misclassified. The samples represented by green and light blue cannot be aggregated, and there are inclusions and dispersions. After passing through other layers of the model and the fully connected layer, each sample is completely separated from the same type of aggregation. It can be shown that the model in this paper can better extract the characteristic information of one-dimensional vibration signal.

#### Bearing fault diagnosis under variable working conditions

**FIGURE 7:**
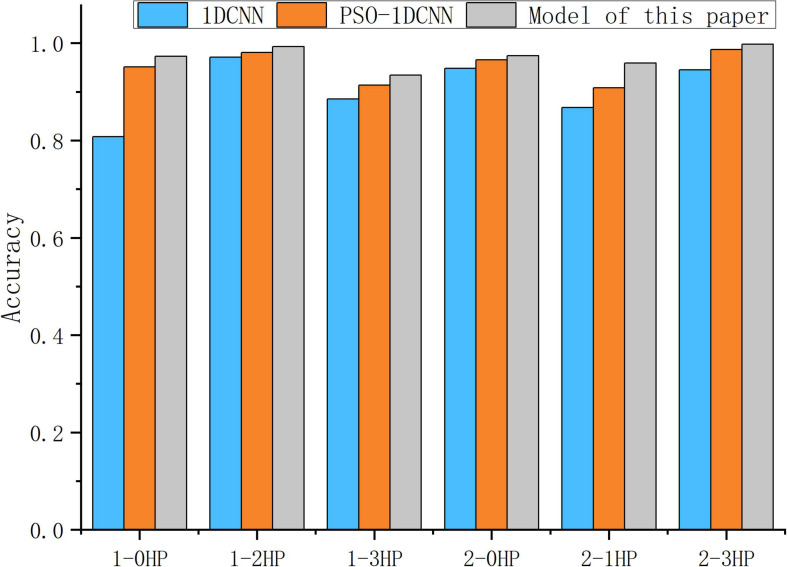
The accuracy of each model under variable working conditions.

In order to verify the generalization ability of the model. In this paper, the models trained under the working conditions 1HP and 2HP are predicted and classified with the test sets of the other three working conditions. Then the 1DCNN model and the PSO-1DCNN model are subjected to the same verification test, and the obtained accuracy is shown in Figure 7. The six variable conditions are 1-0HP, 1-2HP, 1-3HP, 2-0HP, 2-1HP, and 2-3HP. Among them, 1-0HP, 1-2HP, and 1-3HP indicate that the model trained at 1HP is tested at 0HP, 2HP, and 3HP, respectively. 2-0HP, 2-1HP, and 2-3HP indicate that the model trained at 2HP is tested at 0HP, 1HP, and 3HP, respectively. As can be seen from the figure, in the case of six variable working conditions, the model in this paper has improved generalization ability compared with 1DCNN and PSO-1DCNN. The model still retains good accuracy under various working conditions, indicating that the model can solve the problem that the traditional signal processing method cannot maintain the diagnostic accuracy under complex working conditions.

## Conclusion

This paper proposes an improved self-adaptive one-dimensional convolutional neural network for bearing fault diagnosis. Firstly, PSO particle swarm optimization is introduced to adjust the hyperparameters of the network adaptively. Then use the form of long-step large convolution in the first layer to enable the network to generalize to identify temporal features. Furthermore, the network is improved by adding residual connection and global average pooling, so that the model can enhance the generalization ability of the network while maintaining the accuracy. It can be seen from the experimental results that the model can accurately identify bearing faults under different working conditions and the accuracy rate is close to or equal to 100%. At the same time, it can also maintain a good recognition accuracy under variable load conditions. Compared with the traditional 1DCNN and PSO-1DCNN, this model has to achieve better results under different working conditions and variable load conditions.

## Data availability statement

The original contributions presented in this study are included in the article/supplementary material, further inquiries can be directed to the corresponding author.

## Author contributions

XL: experiments, research methods, data processing, and writing the original draft. RWu: resources. RWa, FZ, ZC, and NG: supervision and guided experiments. All authors have contributed to the manuscript and approved the submitted version.
